# Evolving 50–50% bilingual pedagogy in Alberta: what does the research say?

**DOI:** 10.3389/fpsyg.2014.00413

**Published:** 2014-06-17

**Authors:** Rahat Naqvi, Elaine Schmidt, Marlene Krickhan

**Affiliations:** ^1^Werklund School of Education, University of CalgaryCalgary, AB, Canada; ^2^Calgary Board of EducationCalgary, AB, Canada; ^3^Senator Patrick Burns School, Calgary Board of EducationCalgary, AB, Canada

**Keywords:** bilingual pedagogy, cross-linguistic transfer, bilingual learning, Spanish

## Abstract

This paper outlines the provincial frameworks that define the Spanish bilingual program in Alberta, Canada, provides an historical overview of its pedagogic constraints and evolution, and proposes a framework for bilingual pedagogy. The framework is conceptualized from the research evidence of three local case studies, and is based on the centrality of cross-linguistic transfer, in relation to linguistic interdependence and bilingual learning.

## INTRODUCTION

Canada’s evolution as a global voice in language education was facilitated by the 1969 Official Languages Act, and the 1971 articulation of the framework of multiculturalism and bilingualism. Not only did this political underpinning immediately facilitate a coast-to-coast bilingual education movement, i.e., the internationally recognized and well-researched French Immersion program; but also, it has been seminal in the evolution of other significant provincial language and culture initiatives. In this article about a specific provincial scenario, we discuss emerging understandings regarding second language learning pedagogies, as they exist within the current globalized educational milieu.

In Western Canada, in the province of Alberta in particular, an initiative akin to the French Immersion educational concept is alternative bilingual language programs such as English-Chinese, English-German, and English-Spanish (Alberta Education, 2000). Similar to French Immersion, this bilingual model is additive in intent. Its purpose is to develop strong language competencies and literacy in two languages; an objective which is somewhat unique within the scope of North American bilingual programming. There is extensive research (for a complete review see Cummins, 1996, 2001; Genesee and Lindholm-Leary, 2007) describing the various types of bilingual programs prevalent in North America and other countries. The goals of the bilingual programs can differ to a great extent. For example transitional bilingual education, one of the most common forms of bilingual education for minority students in the United States during the past 40 years, aims only to promote students’ proficiency in English (Cummins, 2009). That is to say, when students develop sufficient English language proficiency to follow instructions and write in English, the home language instruction is discontinued and they transition into mainstream classrooms.

In Alberta, the additive bilingual model also emphasizes a range of other educational goals in keeping with current demographic realities and values, such as the trend toward increased choice within public education systems, and the nurturing of global citizenship and intercultural competencies (Holmes, 2008). This approach aligns with the growing national and global trend toward multilingualism and multiculturalism, and is confirmed in Canada’s most recent census, which documents an 11% increase in the population who speak languages other than the dominant language; and a total of 20.6% of Canadians who have a mother tongue other than English or French (The Globe and Mail, 2012). The social dynamics associated with linguistic diversity, the impact of emerging multi-modal technologies, and the central theme of educational policy within economic discourse (Padoan, 2012) result in a radically altered educational landscape that requires both exploration and interpretation.

This paper outlines the provincial frameworks that define the bilingual program, provides an historical overview of its pedagogic constraints and evolution, and proposes a framework for bilingual pedagogy based on theoretical underpinnings and local research evidence.

## EDUCATIONAL FRAMEWORKS THAT PROVIDE THE CONTEXT FOR BILINGUAL PROGRAMS IN ALBERTA

The Spanish bilingual program in Alberta is governed by various key documents, and programming guidelines, outlined below:

### ALTERNATIVE BILINGUAL PROGRAMS

Section 11 of the School Act gives school boards the authority to offer instruction in French or any other language, and Section 21 creates opportunities to learn other languages through alternative programs (Alberta Education, 2000). However, the Alberta Guide to Education states that all programs must offer a minimum of 50% of instructional time in an official language, i.e., English or French (Alberta Education, 2012). Consequently, there is significantly less instructional time allotted to the additional language within a bilingual program than within French Immersion which can dedicate up to 100% of instructional time in French. Bilingual programs operate at 50% English – 50% in the target language at the elementary level, changing to 65% English – 35% in the target language at the junior high level, and 75% English – 25% in the target language at the senior high level, which is determined at the discretion of the school jurisdiction offering the program.

### ALBERTA BILINGUAL PROGRAMS OF STUDY

All grades 1 to 12 bilingual programs share a common framework, rationale and broad objectives as outlined in the respective language arts programs of study. As these language programs with entry points at kindergarten and grade 1, were designed for learners with no previous knowledge of the language, bilingual programs are accessible to all students irrespective of linguistic heritage (Alberta Education, 2005). Functional fluency in the L2 is targeted in each of the four competency areas (reading, writing, listening, and speaking), as well as the capacity for grade appropriate content learning in the L2.

### INTERNATIONAL SPANISH ACADEMY (ISA)

Each alternative bilingual program has evolved somewhat differently and has been influenced by the international relationships that have been nurtured by both the province and each of the school jurisdictions involved. For example, the 26 Spanish bilingual program schools that are unique to the province of Alberta in Canada, have ISA status based on a memorandum of understanding (MOU) for Education Cooperation between the Ministry of Education and Science of the Kingdom of Spain and the Department of Education of the Province of Alberta. This MOU identifies general English-Spanish bilingual program expectations and strategies for advancing international relations and understanding between cultures, including access to visiting international teachers, resources, and professional development (Alberta Education, 2006).

## PEDAGOGIC CONSTRAINTS AND EVOLUTION OF BILINGUAL PEDAGOGY

From the outset, the alternative bilingual language programs in Alberta have been strongly impacted by the traditional assumptions and pedagogy of French Immersion, including strict segregation of learning by language and subject, and by the *maximum exposure hypothesis* implication that only 50% of time in L2 is a deficiency to be managed relative to the learning of the L2 (Cummins, 2001). The notion of creating a dual-language space for explicitly comparing and contrasting languages has not been considered best pedagogical practice, with the view that translation or code switching threatens L2 language growth (Cummins, 2000).

This article highlights challenges faced in light of the current research and emphasizes the need for a pedagogical shift from the *monolingual solitude assumption* to a more flexible approach to language pedagogy (Cummins, 1979, 2005; Creese and Blackledge, 2010; García and Sylvan, 2011; Ó Duibhir and Cummins, 2012). Drawing upon emerging theory on integrated models of language learning, i.e., the strongly supported view of *language-as-a-resource* (Escamilla and Hopewell, 2009) and the *counterbalanced approach* to language learning in content areas (Lyster, 2011; Lyster et al., 2013), researchers discuss the need to inform practice around evolving bilingual pedagogy and literacy acquisition. They highlight the learning potential associated with the *linguistic interdependence principle* (Cummins, 1981, 2001), and recognize *metalinguistic awareness* as being critical in the learning process (Ó Duibhir and Cummins, 2012).

The Spanish bilingual program has encountered a series of unique pedagogical constraints, which are partially rooted in the *monolingual solitude assumption* (Cummins, 1979, 2008; Howatt, 1984). First, by segregating languages of instruction into compartmentalized subject areas, English and Spanish are not integrated into a shared learning space, which could otherwise enhance students’ ability to express their thought processes and to deepen knowledge creation across and between languages (Celic and Seltzer, 2011). Second, when student curiosity is not peaked through relevant cross-curricular work, motivation decreases (Friesen and Jardine, 2009); which in turn conversely impacts language learning (Cummins, 2011; Lyster, 2011). Third, the segregation of languages and subject areas restrict teachers’ abilities to plan inter-disciplinary inquiry projects, and to assess students’ literacy skills considering the entire scope of their linguistic abilities (Cummins, 2005; Escamilla and Hopewell, 2010; Soltero-González et al., 2010).

These pedagogical constraints were further validated at the National Conference of the Association Canadienne des Professeurs d’Immersion in October 2013, in which renowned Canadian bilingual education researchers and advocates, Jim Cummins, Sharon Lapkin, and Fred Genesee engaged in dialog with administrators of Canadian French Immersion education about the need to update the monolingual instruction assumptions upon which French Immersion has operated for nearly 50 years. Empirical studies in recent years (see Dressler and Kamil, 2006 for a review) have consistently promoted the idea of cross-lingual interdependence. Results support Cummins’ longstanding position that learners’ common underlying proficiency be explicitly and strategically developed in order to maximize the cognitive, linguistic and socio-affective capacity of bilingual learners. Cummins posits that “if students are making cross-linguistic connections throughout the course of their learning in a bilingual or immersion program, why not nurture this learning strategy and help students to apply it more efficiently?”(Cummins, 2014).

This perspective is further strengthened by Garcia’s idea of translanguaging based on extensive research with Spanish-English bilingual students and communities living in New York (García, 2009). According to Garcia, the blurred lines between the languages of bilinguals make it important to consider the pedagogical implications and potential cross-linguistic strategies that arise from this interconnectedness. Moreover, within the context of interlanguage awareness, effective instructional strategies promoting two-way transfer across languages in mainstream multilingual classrooms produce clear evidence of how students develop a critical awareness of the social and cognitive functions of languages in their lives (García, 2009).

Although specific to the Spanish bilingual model in Alberta, the proposed framework which promotes general beliefs and values around teaching for transfer across languages is equally valid for other multilingual learning and teaching contexts.

## RESEARCH EVIDENCE THAT INFORMS A CONCEPTUAL FRAMEWORK FOR 50–50% BILINGUAL PEDAGOGY

Between 2011 and 2013 a three part research study was carried out through ongoing collaboration between researchers at the University of Calgary and a Calgary public school district (school-based leaders and teacher practitioners in a primary Spanish bilingual context). In this district, the Spanish bilingual program has grown from approximately 125 students in kindergarten to grade 2 at its inception in 2001, to 3002 students, kindergarten to grade 12 in 2013 (Calgary Board of Education, 2013). To date a vast majority of the students entering the program have been native English speakers who have no previous knowledge of the Spanish language, albeit previously noted national census data shows changing trends in this regard. The continual program growth experienced within the district provoked the need for action research in various components of instructional planning and pedagogy.

The articulated research questions for each study are:

(1) How can the introduction of dual language books (DLB) be used as an instructional strategy in the Spanish bilingual classroom, for strengthening young emerging bilinguals’ explicit awareness of both English and Spanish?
(2) How can authentic task design strengthen cross-linguistic transfer and bilingual identities?
(3) (a) What are the critical principles of an evolving bilingual pedagogy within a holistic learning context? (b) What is the nature of professional learning support needed to leverage such a shift in bilingual pedagogy?

Researchers examined the initial findings of each of the subsequent action research studies in which teachers and students explored the role of cross-linguistic transfer relative to engaging content learning, literacy development in both languages, and pedagogic approaches to second language acquisition. Emerging trends from each of the studies’ key findings were then extrapolated and further described in a proposed framework for bilingual pedagogy.

### PART 1: DUAL LANGUAGE BOOKS IN KINDERGARTEN AND GRADE 1

Dual language books are illustrated books, written in two languages: generally, one language is featured on a page, and the facing page features the other language. By reading these languages in tandem (Sneddon, 2009), it is possible to allow language learners to access a unique fund of knowledge and encourage transfer of conceptual knowledge and skills across languages (Cummins et al., 2005). This study explored the question: how can the introduction of DLBs be used as an instructional strategy in the Spanish bilingual classroom, for strengthening young emerging bilinguals’ explicit awareness of both English and Spanish?

#### Participants and methodology

The research involved 102 students in kindergarten and grade 1, several grade 3 and grade 4 students selected as reading partners and four teachers and parents. Informed consent was obtained for the varying participants, based on district-level ethics protocols. The researchers organized several professional development sessions hosted by the school in which best practices were shared for developing holistic bilingual literacy instruction. As part of these sessions, DLBs were introduced as possible instructional tools and further information was provided to the parent community through letters and meetings. Several goals were identified: (a) to establish targeted instructional strategies for enhancing metalinguistic awareness in pre-readers, (b) to draw in parents as educational partners within the bilingual program, and (c) to encourage older students in the school to participate as readers in the DLB reading program.

Preparatory work to discuss the principles and functions of DLB reading included a 1-hour session for parent readers and mini-sessions for selected grade 3 and grade 4 advanced student readers. Three 20 minute DLB reading sessions, filmed by researchers and assistants, were held weekly and attended by the parent readers. In the first reading, the teacher built vocabulary in Spanish for the text and read a page in Spanish, followed by a parent reading the same passage in English. In the second reading, the parent participant read in Spanish (L2), and the teacher read in English with a focus on explicit metalinguistic awareness. This included vocabulary building, drawing on previous experience with themes, and direct comparisons between the two languages. This was aided by projecting the books using interactive white boards (Smart board technology). In the third reading, the grade 3 and grade 4 students read the text with their younger buddies (kindergarten or grade 1) in six small groups (each group was provided with a copy of the dual language text). The grade 3 and grade 4 students made observations, asked questions, modeled leadership, and interacted with the kindergarten readers about the DLB texts.

All sessions were recorded to capture conversation between all participants on the similarities and differences between languages. Teacher participants, together with other colleagues on the campus, analyzed the video recordings for evidence of metalinguistic awareness. These recordings were also used in professional learning community discussions.

#### Results

When students within the early years bilingual context are provided with strategic mini-readings of DLBs, students, teachers, parents, and student readers have permission to explore commonalities and differences between languages and initiate conversations about language as an object of thought and a resource for enriching interdependent language proficiencies. Further, when languages are displayed on the interactive whiteboard so that comparisons can be made as a class, students develop sophisticated metalinguistic awareness, which supports not only strong second language learning, but also enhances students’ knowledge of their first language. During a reading of a DLB the teacher reflected on the purposeful way that students engaged with both languages, for example one student commented on the different number of letters in the Spanish and English alphabet because the ñs (*ehnyeh*) in Spanish adds another sound and symbol to the Spanish alphabet.

Results taken from video vignette analyses also demonstrated development of leadership skills in student readers as they modeled their bi-literacy and bilingual identity to younger participants. Older students facilitated heightened language awareness and comparison of similarities and differences in text between English and Spanish through a series of noticing exercises. From the professional learning perspective, as a result of the use of DLBs teachers engaged in discussion and exploration of pedagogical strategies to support the growth of metalinguistic awareness, bi-literacy and the emerging bilingual identity of young readers.

As well as increasing parent community involvement in the classroom as a result of volunteering as DLB readers, this process provides the opportunity to coach parents in ways of using bilingual texts to engage readers at home and to further promote metalinguistic awareness in young children. Students were exposed to different accents in Spanish. One teacher described how two mothers from different Hispanic backgrounds had to verify certain words before reading. “So when we were reading the Spanish both of them at different times had to take the turnip book in particular. The lady who was from Mexico had to take and she had to write down words that were written in Spanish because she never heard them before. Same with Señora C from Chile she identified words that she had never seen before.”

### PART 2: CROSS-LINGUISTIC VIDEO LITERACY PROJECT IN GRADE 3 AND 4

Through a single case study the researcher explored the question: How can authentic task design strengthen cross-linguistic transfer and bilingual identities? The aim was to determine what specific aspects of cross-linguistic transfer occurred when engaging in research on the ancient civilization of Peru in L1 (English), and transferring knowledge into the development of a modern Indian Jones story in L2 (Spanish). The video production included creating characters and a plot, writing a script in small groups, and acting in, filming and editing the video recordings in both L1 and L2.

#### Participants and methodology

Over a period of 6 months a multi-grade bilingual teacher, an English speaking professional videographer and 23 grade 3 and grade 4 students participated in this project. Data from the teacher interviews, student learning artifacts, student responses and self-reflections was collected and the results triangulated through the lens of three main aspects of cross-linguistic transfer: conceptual knowledge, linguistic elements, and metacognitive/metalinguistic effects (Ó Duibhir and Cummins, 2012).

#### Results

Analysis of the data revealed the effects of the teacher’s intentionality in bridging conceptual knowledge between the program of studies (Spanish) and the personal, lived experiences of the students (English). Knowledge was created and shared using extensive collaborative dialog, a key precursor to the writing process, which students later worked on together in small groups. The teacher extrapolated key outcomes in the program of studies for both languages, emphasizing the common processes for social and cognitive development. She sought out how the students could build their vocabulary in Spanish, while also learning, building and investigating in English to broaden their knowledge base. This approach positively influenced simultaneous, bilingual literacy growth as well as the integration of learner identity across and between Spanish and English.

Through analyzing student speaking parts of the video project, an active interlanguage phase that includes awareness of content in both languages is evident. The teacher regularly conducted conferences on linguistic elements with small groups of students to build vocabulary, grammatical structures and syntax as the transfer of linguistic elements was weak (verb tenses, word gender, syntax, and possession). These results support earlier work by Verhoeven (1994), in which syntactic functioning transfer is poor. These conferences occurred strategically and in response to expressed student needs. Students also showed sophisticated levels of awareness about these linguistic elements. During student focus group interviews and in reflection journals, they commented on how their language had changed through more exposure to explicit instruction in sentence structure and syntax.

Salient to the process of inquiry was the ongoing feedback that the teacher requested of the students in their own, and each other’s learning, including evaluating one another’s Spanish scripts, and assessing aspects of oral language production through student-created rubrics. A final reflection piece was given to students at the conclusion of the project with questions posed in English, such as: *what new skills did you learn?* and, *how will these skills help you in your life, or in another project?* The teacher described the metacognitive and metalinguistic awareness processes as being integral to the building of self-awareness throughout content and language learning.

Results of the case study confirm that students’ engagement through meaningful, relevant inquiry is the most significant indicator of their level of engagement with the second language. Engagement is best supported through multi-disciplinary tasks which intentionally enlist both L1 and L2 in the creation of cross-linguistic knowledge transfer and ultimately enhance bi-literacy and growth of bilingual identity in learners. Multi-disciplinary tasks also facilitate strategic engagement of metalinguistic awareness and explicit development of features of the L2.

### PART 3: PROFESSIONAL LEARNING JOURNEY OF SPANISH BILINGUAL PROGRAM TEACHERS

The multi-faceted goals of this provincially mandated additive bilingual model combined with the lack of an articulated pedagogy for this model, produced a complex pedagogic challenge for bilingual program teachers and leaders. They attempted to shift their professional practice to explore bilingual pedagogy, and simultaneously to identify, nurture, and sustain the professional learning process needed to advance this exploration. Consequently this study addressed a two-part question: (a) what are the critical principles of an evolving bilingual pedagogy within a holistic learning context? and, (b) what is the nature of professional learning support needed to leverage such a shift in bilingual pedagogy?

#### Participants and methodology

Over a period of three school years a campus team of 25 kindergarten to grade four Spanish-English bilingual program teachers participated in the study. Within the school’s Professional Learning Community (PLC) structure, members of the team collaborated in designing authentic inquiry-based learning tasks across the two languages (L1 and L2), exploring the tasks with students, and then collecting, sharing and analyzing artifacts and feedback with colleagues in the PLC context. The bilingual pedagogic challenges and strategies and the collaborative professional learning needs were tracked through several cycles of professional inquiry during the 2010–2011 school year. Qualitative data was gathered using the tools of individual teacher questionnaires, a teacher focus group, and PLC reflections. As well, at the end of the year, four grade 3 and grade 4 students were interviewed to validate and further inform teacher perceptions about this experience. During the next two school years 2011–2012 and 2012–2013, two individual follow-up teacher video interviews were conducted, and additional professional learning community questions were generated during a staff retreat. This additional data contributes to building a longitudinal perspective about this professional learning experience.

#### Results

Through the eyes of practicing bilingual teachers and corroborated by student voice, this study tracks shifts in practice from traditional teacher-directed language learning to increased design-for-learning that capitalized on activation of cross-linguistic transfer. Teachers cited examples of increasing sophistication in critical thinking and increased levels of intellectual engagement as students made conceptual connections and applied skills across languages. One teacher describes both languages as the tools that support the content, which is the star of each conversation. She continued to say that sometimes students are not aware that they are asking a question in Spanish. At the same time, students articulated approaches and strategies for dealing with language related challenges, which demonstrates increasing awareness of their personal control of learning. For example one student commented on the ease of learning when connections existed between the classroom languages, and another student articulated how her strategy to independently borrow and read library books in L2 has positively impacted her Spanish pronunciation and her confidence to take risks in learning. From the perspectives of teachers and students, engagement in learning was being activated by the discovery of meaningful connections across content and across languages. Further, engagement facilitated by cross-linguistic transfer contributed positively to the growing bilingual identity of students, as was demonstrated by the increased amount of natural flow between languages.

Teachers quickly identified pedagogic questions and challenges relevant to a shift that focuses on cross-linguistic learning in a holistic environment. This includes questions about task design and strategies for cross-linguistic transfer, appropriate interactive classroom structures for L2 practice and feedback, and principles of instructed language learning as they pertain to the effective role of L1 and access to extended time to L2. Teachers were adamant that access to expert knowledge on current pedagogy and collaborative exploration of professional learning environments including, observation, peer coaching and resource development are critical to the effective evolution of a 50–50% bilingual pedagogy.

To summarize, this professional inquiry perspective data supports several key findings: (1) when students learn in holistic contexts, there is strong evidence of cross-linguistic transfer, as well as growing metalinguistic awareness and an evolving bilingual identity, (2) teachers identified their need for articulating appropriate second language acquisition strategies within this context and for facilitating student collaboration environments, (3) teachers identified their need for access to expertise on second language pedagogic approaches, and for regular collaborative inquiry and peer-coaching opportunities.

## FRAMEWORK FOR BILINGUAL PEDAGOGY: THE ROLE OF CROSS-LINGUISTIC TRANSFER

**FIGURE 1:**
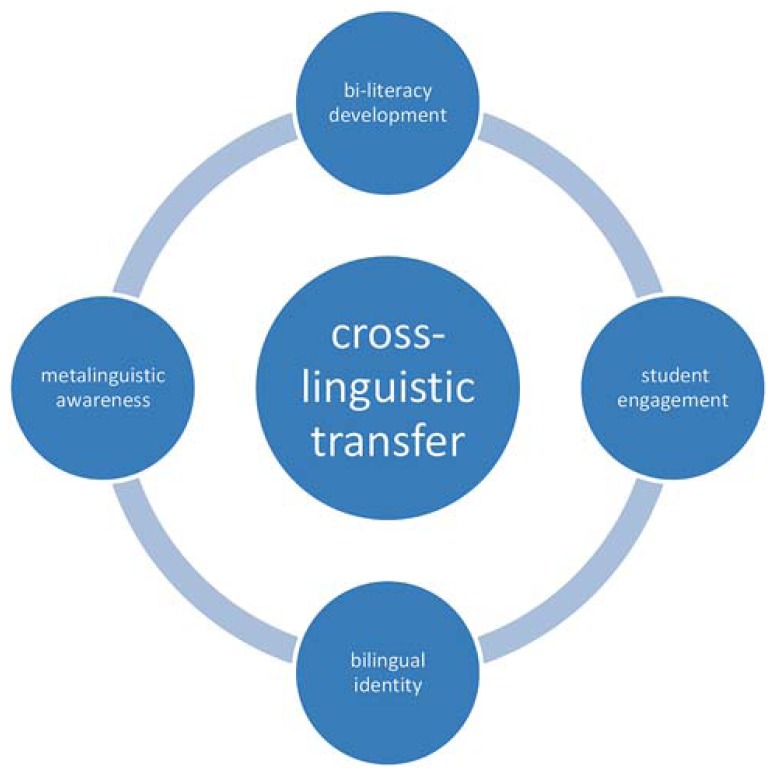
**Conceptual framework for 50–50% bilingual pedagogy, diagramming the centrality of cross-linguistic transfer in relation to linguistic interdependence and bilingual learning**.

As a result of ongoing research and collaboration between the school jurisdiction and the University of Calgary, the authors propose a conceptual framework representing an evolutionary shift in pedagogical practices in elementary bilingual schools. While the principles highlighted below are specific to one elementary Spanish bilingual school, they provide relevance to other bilingual settings in Alberta as well. In the framework shown in **Figure 1** the researchers situate cross-linguistic transfer, rooted in the *principle of linguistic interdependence*, at the center of bilingual learning. Cross-linguistic transfer at the center facilitates a flexible, reciprocal and dynamic interplay between content, language and the student learning experiences. When viewed as a theory of action, the framework proposes that when learning in the bilingual context focuses on two-way transfer across languages, then learners will develop stronger metalinguistic awareness and enhanced bi-literacy skills; while experiencing greater student engagement and therefore nurturing bilingual identities.

Our data demonstrates that as teachers and students involved in the three action research studies explored the potential of cross-linguistic transfer in various learning contexts, they experienced extensive examples of linguistic interdependence and related learning effects. Below, in the words of teachers and students, we present examples that illustrate the specific elements of this conceptual framework in relation to these experiences.

### BI-LITERACY DEVELOPMENT

In the professional learning study teachers repeatedly commented that students were trying strategies without being asked, and transferring the literacy skills from language to language. Comments included: “Students are independently transferring English story telling skills to relevant Spanish language contexts”; and “students spontaneously switched to debating in Spanish during a news debate started in English.” Another teacher commented on her own uncertainty as to whether she had taught students the structuring of informative texts in Spanish or in English, she concluded by saying that ultimately it did not matter because meaningful learning was happening in both languages and the knowledge and skills taught or practiced in one language were soon applied in other relevant learning contexts. In the DLB study teachers provided similar examples where students were building a deeper knowledge of the two languages: “It was really fun and delightful to see one of my students,” “Well Señorita M. those two sounds are the same when you hear them but they’re written differently in the book.”

### BILINGUAL IDENTITY

Teachers and students provided many examples of personal engagement in either language when students participated in meaningful tasks and in a risk-supported environment. One student described his experience: “sometimes it just happens (in the other language), it comes to mind and I switch.” A student participating in the video literacy project said: “We came up with all the ideas in our head in English, and it just came out in Spanish. There was no – like – How do you fit this word in here?” Another student shared: “at home in the middle of dinner, I speak some in Spanish!” Children as young as 4 years old were negotiating varying colloquial terms for “school bus” in varying Spanish-speaking countries, discussing the “wua-wua” in Cuba and the “camion” in Mexico.

### METALINGUISTIC AWARENESS

As the study progressed students were able to articulate specific aspects of their own languaging which had changed since being in the bilingual program. For example when reflecting on the video literacy project a student said: “When I was in kindergarten I used to say,” “Yo gusta.” Now that I am in grade 4, there is no such thing. Now it’s “A mi me gusta.” Another student shared: “In kindergarten I couldn’t say anything, now I can say paragraphs and sentences, I can finally write in Spanish.” They reflect on how they spoke initially and on how their understanding of grammatical elements and usage had grown. As such, they recognized their own growth along the interlanguage continuum. Another student contrasted the two languages: “the easiest part of learning Spanish is the sounds, the way you pronounce is the way you read it, but in English there are funny sounds; you can guess what the word is in English.”

### STUDENT ENGAGEMENT

Authentic tasks are at the heart of student engagement and when cross-linguistic transfer is employed to deepen the learning experience, heightened student engagement and more self-directed learning follows. As one teacher commented: “They didn’t ask for help, they use their English skills…”; and from a students’ perspective: “Everything is connected in our class [topics in English and Spanish], it helps us if it’s connected.” One teacher described the level of ownership for learning that she was now experiencing with her students: “They told me what to write on their report cards and once they did that they owned it, they are engaged and it leads to the adjustment cycle.” Within the context of the DLB readings grade 3 and 4 students read to the grade 1 students in Spanish and asked them to identify linguistic differences between English and Spanish. “Today, in our last reading one group with Jessica’s kids were umm Señora, we are, we have challenged these grade ones today, we are challenging them to find five differences.” Within the video literacy project, the teacher described her planning processes in this way: the students all began to think about how they could tie in the Incan [people] with a story that sparked their interest in unraveling ancient clues. We discussed what types of personalities and characters they wanted to include. And we held onto that piece that excited them in the theaters about Indiana Jones, and with that they began to create their own story. I began to think, most of the background information was conducted in English. “How could we take that interest and start to transform it, and to build their Spanish vocabulary around it?”

We specifically refer to the Ó Duibhir and Cummins (2012) report, in which extensive research highlights similar evidence to support the view that:

“literacy-related skills and knowledge can be transferred across languages. When teachers encourage this transfer explicitly they make learning more efficient for the learners and reinforce effective learning strategies.” (p. 12).

This position is reinforced and mandated in the Alberta bilingual programs of study, wherein the rationale explicitly addresses cross-language competence, stating that many of the first language skills of learners are transferable within the stated bilingual programming context and that in acquiring a new language, these skills can also be transferred to the first language (Alberta Education, 2005). This document further identifies effective bilingual learning environments as those where there is a significant relationship between various subject area experiences and where connections to prior knowledge and experience are made (Alberta Education, 2005).

## DISCUSSION: CONCLUSION AND RECOMMENDATIONS

Referring back to Ó Duibhir and Cummins (2012) and to the rationale in the Alberta bilingual programs of study, the researchers advocate for a pedagogy building on connections across languages (in this specific example Spanish and English). Our results provide powerful examples of flexible approaches to bilingual pedagogy that foster higher levels of literacy engagement in both languages, as well as other relevant learning effects as described in **Figure 1**. “When we free ourselves from exclusive reliance on monolingual instructional approaches, a wide variety of opportunities arise…that acknowledge the reality of, and strongly promote cross-linguistic transfer (Cummins, 2007).

Through our findings we invite practitioners to reflect on the proposed framework as a stepping stone to evolving a collaborative pedagogy with cross-linguistic transfer at the center of this work. Our results provide a strong argument for practitioners and researchers to address the following recommendations:

(1) Allow for flexibility in scheduling so that inter-disciplinary and inter-linguistic learning facilitate cross-linguistic transfer; ultimately supporting all elements in the framework.
(2) Participate in further action research regarding current bilingual pedagogy that is tailored to each specific learning context and the potential programming similarities and differences in each [e.g., elementary years (kindergarten to grade 6) 50% English-50% Spanish vs. junior high (grade 7 to grade 9) 65% English-35% Spanish], as well as further research regarding metacognitive and metalinguistic awareness in this cross-linguistic learning environment.
(3) Research approaches for explicitly addressing the second language learning component within this bilingual context.
(4) Negotiate professional learning networks to participate and collaborate in action research to further explore the dynamics of inter-linguistic task design, with specific attention to feedback and practice in L2, and to address controversial elements such as translanguaging and translation in the bilingual classroom, while specifically outlining the role and purpose for language usages in task design.
(5) Support recruitment and professional learning that assures an aligned vision of appropriate pedagogy for the bilingual context.

## Conflict of Interest Statement

The authors declare that the research was conducted in the absence of any commercial or financial relationships that could be construed as a potential conflict of interest.
